# A nomogram of suicidal ideation among men who have sex with men in China: Based on the integrated motivational-volitional model of suicidal behavior

**DOI:** 10.3389/fpubh.2022.1070334

**Published:** 2022-12-22

**Authors:** Chen Xu, Zuxin Wang, Shangbin Liu, Hui Chen, Yingjie Chen, Danni Xia, Yufei Chen, Huifang Xu, Fan Hu, Ying Wang, Yong Cai, Jianyu Chen

**Affiliations:** ^1^Clinical Research Unit, Tongji University Affiliated Shanghai Pulmonary Hospital, Shanghai, China; ^2^School of Public Health, Shanghai Jiao Tong University School of Medicine, Shanghai, China; ^3^College of Public Health, Shanghai University of Medicine and Health Sciences, Shanghai, China; ^4^Hongqiao International Medical Research Institute, Shanghai Jiao Tong University School of Medicine, Shanghai, China

**Keywords:** men who have sex with men, suicidal ideation, the integrated motivational-volitional model of suicidal behavior, nomogram, prediction model

## Abstract

**Introduction:**

Men who have sex with men (MSM) are a high-risk group for suicide, with a much higher prevalence of suicidal ideation (SI) than the general population and male population. The aim of this study was to explore the risk factors influencing the development of SI and to develop and validate a nomogram among MSM.

**Methods:**

A cross-sectional study was conducted in 915 MSM from Shanghai, Shenyang, Shenzhen and Kunming, China using the snowball sampling method. The integrated motivational-volitional (IMV) model of suicidal behavior was used as a theoretical framework to collect different potential influencing factors of SI including diathesis-environment-life events factors and psychosocial factors. The risk factors of SI were screened by logistic regression analysis, and a nomogram for predicting SI were developed. Model properties including discrimination, calibration and decision curves were evaluated to validate the prediction model.

**Results:**

882 MSM were included in the statistical analysis, with a lifetime prevalence of SI of 34.4% (303/882). Logistic regression analysis showed that age group, sexual orientation disclosure, high-risk sexual behavior, entrapment, defeat and interpersonal needs were associated with SI. A nomogram was constructed based on the above six predictors. AUC values of ROC for prediction model were 0.761 (0.641–0.770) and 0.754 (0.565–0.822) in the training set (*n* = 662) and validation set (*n* = 220), respectively. And there was no statistical difference of the AUC values between the two sets (*P* > 0.05). The calibration plots of the prediction model in both sets fit well with the ideal model (*P* > 0.05). The decision curves demonstrated that the threshold probability of prediction model in training set was 1–85%, whereas in validation set was 1–63%.

**Conclusions:**

The lifetime prevalence of SI among Chinese MSM is high. The nomogram can serve as a useful tool to predict the development of SI among MSM. Defeat, entrapment and interpersonal needs, as significant predictors of SI, can be measured to identify SI in advance. Early assessment of SI and the enhancement of psychosocial interventions are important to prevent suicide-related behaviors. Future studies could incorporate more variables of interest to refine the prediction model to better guide behavioral and psychological intervention strategies among MSM.

## 1. Introduction

Men who have sex with men (MSM) include gay men, bisexual men, heterosexual men, and transgender men (1). MSM, as a group of sexual minorities, are not legally recognized and socially supported because of their particular sexual orientation or same-sex sexuality. Stigmatized environment exposes MSM to discrimination, prejudice, social isolation and exclusion, stigma, abuse, violence and mental health disorders, etc (2, 3). As a result, MSM represent a vulnerable population with respect to suicide-related behavior which is a leading cause of death and disability and has become a serious threat to their health (4). It is essential to reduce the negative outcomes and the burden of disease through timely identification of suicide-related behavior and interventions to prevent suicide.

The pathways to suicide are complex. Suicidal behavior goes through three phases: suicidal ideation (SI), suicide attempt (SA) and completed suicide. SI is defined as thinking about, considering or planning for suicide. SA is an intentional, self-inflicted, life-threatening act that results in bodily injury but not death (5). SI is considered as a precursor to suicidal behavior and an important predictor of suicide attempts and deaths (6). The people who expressed SI have higher risk of suicide than those without SI with a relative risk ratio (RR) of 4.17 [95% confidence interval (CI): 3.29-5.27] (7). Mathy found that MSM in South America, North America and Asia had 7.5, 2.1 and 2.9 times the risk of SI than heterosexual males, respectively (8). A systematic review reported a lifetime prevalence of SI among MSM in China was 21.2% (95% CI: 18.3–24.5%), much higher than the prevalence of the general population (*P* = 3.9%) (9) and male population (*P* = 6.4%) (10, 11). Given the high prevalence of SI and the important role of SI in the suicidal process, the assessment of SI is imperative among MSM.

The development of SI and transition from ideation to attempt should be viewed as distinct processes with different predictors according to the “ideation-to-action” framework proposed by Klonsky and May (12). Therefore, Rory O'Connor proposed the integrated motivational-volitional model (IMV) of suicidal behavior and attempted to explain this issue (13). The IMV model, as a comprehensive theoretical model, integrates four theoretical models of suicide including diathesis-stress model, the theory of planned behavior (TPB), cry of pain theory of suicide and differential activation hypothesis (14–16).

**Figure 1:**
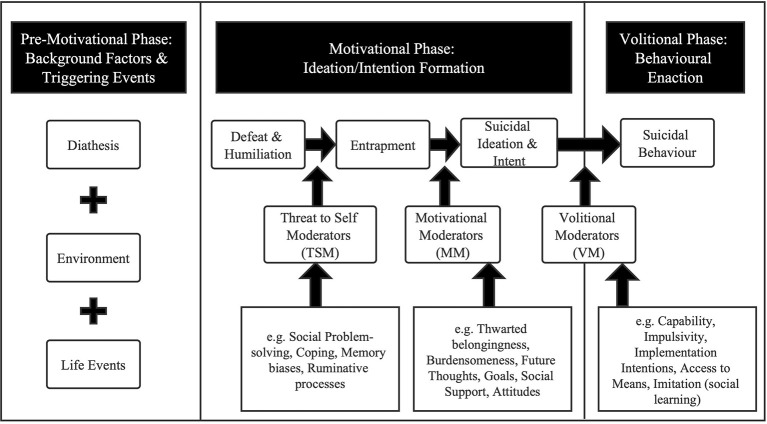
The IMV model of suicidal behavior (18).

The IMV model consists of three phases and delineates the detailed pathways to suicidal ideation and behavior. The pre-motivational phase describes the biopsychosocial context in which suicidal ideation and behavior may emerge. Vulnerability factors combined with diathesis–environment–life events provide the backdrop for the generation of SI. The motivational phase describes the ideation/intention formation focusing on the psychosocial processes that may lead to the emergence of suicidal ideation and intent. The framework posits that SI begins with feelings of defeat (a sense of failed social struggle, losing social status, powerlessness or missing personal goals) and humiliation, and a sense of entrapment (there is no perceived escape) is the proximal predictor of SI (17). Thwarted belongingness (TB) and perceived burdensomeness (PB), as two important motivational moderators (MMs), moderate the association between the entrapment and SI. The volitional phase outlines volitional moderators (VMs) that govern the transition from SI to suicide attempts/death (16) (Figure 1).

According to the IMV model, the potential risk factors of SI among MSM can be grouped into different phases. In the pre-motivational phase, some background factors such as age, race, education, employment status, income, and marital status may influence the development of SI (6). Sexual orientation is an important feature of MSM and there is strong evidence of association between suicide risk and bisexuality or homosexuality in males (19). A proportion of MSM choose to conceal their sexual orientation to avoid prejudice or discrimination. MSM are prone to high-risk sexual behaviors such as unprotected anal sex or multiple partners (20). Meanwhile, MSM have a higher rate of substances use like tobacco use, alcohol use and drug use than the general males (21). All these behaviors have the potential to act as triggering events leading to suicide-related outcomes. Numerous studies have shown that psychological factors such as hopelessness, depression, anxiety and loneliness are all positively associated with SI (22–24). But more social support received from families and friends can protect MSM from the risk of suicide (25). People with SI tended to experience higher levels of defeat and entrapment than people without SI (26). Thwarted belongingness and perceived burdensomeness, collectively known as interpersonal needs, are considered to be the psychological states in closest proximity to SI. However, the roles of entrapment, defeat and interpersonal needs in the development of SI among MSM remain to be explored.

There are quite a few studies that apply various methods including logistic regression analysis, structural equation model and syndemic theory to explore the prevalence and influencing factors of SI among MSM (4, 10, 23, 27). At present, the prediction models are mostly used for the diagnosis and prognosis of clinical diseases, and they have also been gradually applied in the field of behavioral psychology. Nomogram is widely used as a reliable tool to predict individual risk in reported outcomes by integrating diverse risk factors (28). Some studies have applied nomogram to predict SI and SA among depressed population (29, 30), adolescents (31), patients with cancer (32), showing good fitness to help implement individual strategies. However, no studies have applied the prediction model to predict the probability of SI among MSM. Therefore, this study aimed to use the IMV model as the theoretical framework to collect the diathesis-environment-life events and psychosocial factors that may affect the SI of MSM. Then we developed a nomogram using the potential predictors of SI, and evaluated its predictive ability.

## 2. Methods

### 2.1. Study population and eligible criteria

A cross-sectional study was conducted in four cities in China (Shanghai; Shenyang, Liaoning Province; Shenzhen, Guangdong Province; Kunming, Yunnan Province). The participants were MSM who met the following inclusion criteria: (1) Over 18 years old; (2) having sex with men in the past 6 months; (3) signing the informed consent and indicating willingness to cooperate. The exclusion criteria were inability to express true feelings or to complete the questionnaire for various reasons.

### 2.2. Recruitment and study procedure

The non-public and hidden state of MSM made it difficult to conduct a random sampling survey, so this study used a snowball sampling method to recruit the participants (33). In collaboration with the local non-governmental organizations and Center for Disease Control and Prevention, 5–10 eligible MSM were selected as initial “seeds” in each district. These “seeds” needed to meet the following criteria: (1) fitting the definition of MSM in this study; (2) being prestigious and trusted in the MSM circle; (3) having high social network size and diverse social connections in the MSM circle; (4) having the ability and enthusiasm to persuade others to participate in the study. Then they were required to recruit more eligible subjects with the same sociocultural background as the second subgroups. The subgroups also summoned other familiar companions, and so on, to create a snowball effect and reach saturation.

It was found that the lifetime prevalence of SI among MSM in China was about 26.01% (34). Using alpha of 0.05 and a relative error for sampling 0.05, the sample size calculated by the PASS 15.0 software was 314. Taking into account the non-probability sampling and a 20% non-response rate, the final sample size was set to be 785.

### 2.3. Data collection

Before the investigation, unified trainings were conducted to make investigators understand the survey content, methods and precautions, and reach a consensus on the questionnaire. The investigation process was divided into two parts. First, the investigators explained the purpose, process and significance of the research to the participants in detail, and obtain their informed consent. The participants were then asked to independently complete a self-administered questionnaire in a separate room. Each questionnaire took approximately 30–40 minutes to complete. After the questionnaires were completed, the investigators performed completeness checks and logic checks to ensure the data accuracy. Certain financial subsidies were be provided to the participants.

### 2.4. Questionnaire design

#### 2.4.1. Diathesis-environment-life events

Background factors included age group, education level, marital status, monthly income, local residence, and sexual orientation and sexual orientation disclosure. Triggering events included tobacco use, alcohol use, drug use, high-risk sexual behavior, verbal harassment, and physical assault.

#### 2.4.2. Psychosocial factors

##### 2.4.2.1. Entrapment

The entrapment scale (ES) was developed by Gilbert and Allan to assess an individual's feeling of entrapment. The scale has 16 items. The options for each item include “not at all,” “a little bit,” “moderately,” “quite a bit” and “extremely,” corresponding to scores of 0 to 4. The total scale score ranges from 0 to 64, with higher score indicating a higher level of entrapment (17). The Chinese version of the ES has been validated with good reliability among MSM (35).

##### 2.4.2.2. Defeat

The defeat scale (DS), designed by Gilbert and Allan, was applied to assess perception of defeat over the past 7 days. The scale consists of 16 items and it is a 5-point scale ranging from 0 (never) to 4 (always). The total score varies from 0 to 64 points. The more likely one feels defeated in daily life, the higher the scale scores (17). The reliability and validity of Chinese version of the DS have been evaluated in various populations (36, 37).

##### 2.4.2.3. Interpersonal needs

Van Orden designed the Interpersonal needs questionnaire (INQ) to reflect respondents' perceived burdensomeness and thwarted belongingness in the past week (38). The scale consists of 15 items. Each item is rated on a scale of 1 (not at all true for me) to 7 (very true for me). Higher scores represent higher levels of unsatisfactory interpersonal needs. The Chinese version of the INQ has good psychometric properties among Chinese workers (39).

##### 2.4.2.4. Social support

The level of social support from family, friends and other key contacts was evaluated by Multiple Scale of Perceived Social Support (MSPSS) (40). The scale contains 12 items and the points of each item scale 1 to 7 indicating “very strongly disagree” to “very strongly agree.” The total score is obtained by summing the scores of each item, ranging from 12–84. The higher the total score, the higher the level of social support felt by the individual. The Chinese version of MSPSS have been proven to be valid and reliable (41).

##### 2.4.2.5. Suicidal ideation

With reference to previous studies, the respondents were asked the specific question to assess lifetime suicidal ideation (“Have you ever considered suicide?”), with options including “yes” and “no” (10, 42).

### 2.5. Ethics

This study has been approved by the Ethics Committee of the School of Public Health and Nursing, Shanghai Jiao Tong University School of Medicine (ethics approval number: 2016013). The entire research process guaranteed the right of the objects. All participants signed the written informed consent. The whole process of the survey and data analysis was anonymous to fully protect the privacy of the participants.

### 2.6. Statistical analysis

Normally distributed variables were described by mean ± standard deviation (SD), skewed variables were described by median (interquartile range, IQR), and categorical variables were described by frequency (percentage). To explore the validity and reliability of the scales, we performed Kasier-Meyer-Olkin (KMO) test, Barlett's test of sphericity and principal component analysis to explore the construct validity. Cronbach's α and Spearman-Brown coefficients were used to evaluate the internal consistency reliability and split-half reliability, respectively. Usually, 0.70 is recommended as a minimum standard for reliability (43).

Univariate and multivariate logistic regression analyses were used to screen the risk factors of SI. *P* < 0.05 indicates statistical significance. Then the subjects were randomly divided into training set and validation set with the ratio of 3:1. The chi-square test or non-parametric test were used to explore whether the distribution of variables in two sets. We established a nomogram using the predictors selected from logistic regression analysis in the training set.

For the validation of the nomogram, we calculated discrimination, calibration and decision curve analysis (DCA) based on the training set and validation set. Discrimination can be measured by the receiver operating characteristic (ROC) curve and the area under the curve (AUC). AUC > 0.9 indicates that the model has great discrimination, a value between 0.7–0.9 indicates good discrimination, and a value between 0.5–0.7 indicates poor discrimination (44). The calibration was presented by the calibration plot and Hosmer-Lemeshow goodness-of-fit test. The closer the calibration plot is to the 45-degree diagonal line, the better the degree of calibration (45–47). The decision curve, which consists of threshold probability and net benefit (NB), was used to show the benefit of the prediction model. The larger the threshold probability and the net benefit value, the better the practicality of the model (48, 49). All statistical analysis methods were implemented with the R software (version 3.6.2; https://www.R-project.org).

## 3. Results

A total of 915 valid questionnaires were collected in four cities (312 in Shanghai, 282 in Shenyang, 199 in Shenzhen and 122 in Kunming), which met the minimum sample size requirement. One case with a widowed marital status and 32 cases with an unclear sexual orientation were excluded. Finally, 882 MSM were included in the statistical analysis (297 in Shanghai, 277 in Shenyang, 189 in Shenzhen and 119 in Kunming).

### 3.1. Descriptive analysis and the associations between different factors and SI

A total of 303 reported ever considering suicide among 882 MSM, and the lifetime prevalence of SI was 34.4%. The average age was 32 years with a standard deviation of 8.46 and 83.5% were younger than 40 years. More than half (66.0%) had an educational level above high school. 82.9% were unmarried and 27% earned less than an average of 3,000 yuan each month. 76.8% were not local residents. 619 (70.2%) reported their sexual orientation as homosexual and 59% did not conceal their sexual orientation. Univariate logistic regression analysis revealed that age group, education level, monthly income and sexual orientation disclosure may be associated with SI. The detailed information was shown in Table 1.

**Table 1:** Background factors and their associations with suicidal ideation (*n* = 882).

	***n* (%)**	**Had SI *n* (%)**	**Prevalence of SI (%)**	**OR (95%CI)**
**Age group (years)**				
≤29	371 (42.1)	146 (48.2)	39.35	1
30–39	365 (41.4)	121 (39.9)	33.15	0.76 (0.57–1.03)
40–49	100 (11.3)	29 (9.6)	29.00	0.63 (0.39–1.12)
≥50	46 (5.2)	7 (2.3)	15.22	0.28 (0.12–0.64)^*^
**Education level**				
Less than Junior high school	134 (15.2)	28 (9.2)	20.90	1
High school	166 (18.8)	57 (18.8)	34.34	1.98 (1.17–3.35) ^*^
Uni. /tech./prof	582 (66.0)	218 (71.9)	37.46	2.27 (1.45–3.55) ^*^
**Marital status**				
Married	110 (12.5)	31 (10.2)	28.18	1
Unmarried	731 (82.9)	259 (85.5)	35.43	1.40 (0.90–2.18)
Divorced	41 (4.6)	13 (4.3)	31.71	1.18 (0.54–2.58)
**Monthly income (RMB)**				
≤3000	238 (27.0)	90 (29.7)	37.82	0.90 (0.63–1.28)
3001–6000	303 (34.4)	107 (35.3)	35.31	0.86 (0.59–1.26)
6001–12000	238 (27.0)	82 (27.1)	34.45	0.50 (0.30–0.85) ^*^
≥12001	103 (11.7)	24 (7.9)	23.30	
**Local residence**				
No	677 (76.8)	231 (76.2)	34.12	1
Yes	205 (23.2)	72 (23.8)	35.12	1.05 (0.75–1.45)
**Sexual orientation**				
Heterosexuality	17 (1.9)	5 (1.7)	29.41	1
Homosexuality	619 (70.2)	233 (76.9)	37.64	1.45 (0.50–4.16)
Bisexuality	246 (27.9)	65 (21.5)	26.42	0.86 (0.29–2.54)
**Sexual orientation disclosure**				
Yes	520 (59.0)	202 (66.7)	38.85	1
No	362 (41.0)	101 (33.3)	27.90	0.61 (0.46–0.81) ^*^

* P < 0.05; OR, odds ratio.

There were 603 (68.4%) and 613 (69.5%) who reported no tobacco use in the past 30 days and no alcohol consumption before sexual behavior, respectively. More than half (51.6%) had drug use, especially rush poppers. 568 (64.4%) had high-risk sexual behavior (unprotected anal sex or multiple partners) in the past 6 months. 19.7% and 6.3% reported having been verbally abused or physically assaulted by others for their same-sex sexuality, respectively. Univariate logistic analysis reported that high-risk sexual behavior may be positively associated with SI (Table 2).

**Table 2:** Triggering events and their associations with suicidal ideation (*n* = 882).

	***n* (%)**	**Had SI *n* (%)**	**Prevalence of SI (%)**	**OR (95%CI)**
**Tobacco use**				
No	603 (68.4)	212 (70.0)	35.16	1
Sometimes	126 (14.3)	41 (13.5)	32.54	0.89 (0.59–1.34)
Often/everyday	153 (17.3)	50 (16.5)	32.68	0.90 (0.61–1.31)
**Alcohol use**				
No	613 (69.5)	203 (67.0)	33.12	1
Sometimes	251 (28.5)	91 (30.0)	36.25	1.15 (0.85–1.56)
Often/everyday	18 (2.0)	9 (3.0)	/	2.02 (0.79–5.17)
**Drug use**				
No	427 (48.4)	144 (47.5)	33.72	1
Yes	455 (51.6)	159 (52.5)	34.95	1.06 (0.80–1.39)
**High–risk sexual behavior**				
No	314 (35.6)	89 (29.4)	28.34	1
Yes	568 (64.4)	214 (70.6)	37.68	1.53 (1.13–2.06)^*^
**Verbal harassment**				
No	708 (80.3)	238 (78.5)	33.62	1
Yes	174 (19.7)	65 (21.5)	37.36	1.18 (0.83–1.66)
**Physical assault**				
No	826 (93.7)	281 (92.7)	34.02	1
Yes	56 (6.3)	22 (7.3)	39.29	1.26 (0.72–2.19)

* P < 0.05; OR, odds ratio.

The results of the KMO test and the Bartlett's test of sphericity indicated the scales were suitable for factor analysis. The principal component analysis showed that 1, 2, 3, and 2 common factors were extracted from ES, DS, INQ, and MSPSS, respectively. The Cronbach's α coefficients and Spearman-Brown coefficients of four scales were greater than 0.8, indicating good internal consistency reliability and split-half reliability (Table 3). The median (IQR) of the ES, DS, MSPSS, INQ were 23 (18), 25 (19), 61.5 (23.25), and 42 (23), respectively. All four psychosocial factors including entrapment, defeat, social support and interpersonal needs were associated with SI. Higher levels of entrapment and defeat, lower levels of social support, and unmet interpersonal needs were more likely to increase the risk of SI (Table 4).

**Table 3:** Validity and reliability tests of the scales (*n* = 882).

**Scales**	**KMO test**	**Bartlett's test of sphericity (*p*-value)**	**Components extracted**	**Sums of squared % of variance**	**Cronbach's α coefficient**	**Spearman–Brown coefficient**
ES	0.97	<0.001	1	69.33	0.97	0.98
DS	0.96	<0.001	2	75.75	0.93	0.96
INQ	0.89	<0.001	3	70.51	0.95	0.88
MSPSS	0.94	<0.001	2	76.79	0.86	0.97

**Table 4:** Psychosocial factors and their associations with suicidal ideation (*n* = 882).

**Psychosocial factors**	**Range**	**Mean**	**SD**	**Median**	**IQR**	**OR (95%CI)**
Entrapment	0–64	24.9	14.58	23	18	1.06 (1.05–1.07)^*^
Defeat	0–64	25.88	13.22	25	19	1.07 (1.05–1.08)^*^
Interpersonal needs	15–99	41.97	15.67	42	23	1.04 (1.03–1.05)^*^
Social support	12–84	60.06	15.41	61.5	23.25	0.97 (0.96–0.98)^*^

* P < 0.05; OR, odds ratio.

### 3.2. Development and verification of nomogram

Multivariate logistic regression analysis was conducted using the statistically significant variables in the univariate logistic regression analyses. Six potential risk factors were screened out and used in the construction of the nomogram. The results showed that the participants over the age of 50 were at a lower risk of developing SI compared to those under the age of 30 (OR = 0.27). Subjects who disclosed their sexual orientation had a 1.58-fold increased risk of developing SI compared to those who concealed sexual orientation. MSM who had high-risk sexual behavior were 1.47 times more likely to develop SI than those who didn't have high-risk sexual behavior. The higher the scores of the ES, DS and INQ, the higher the risk of SI with the OR values of 1.03, 1.03, and 1.02, respectively (Table 5).

**Table 5:** Risk factors of SI selection using multivariate logistic regression (*n* = 882).

**Variables**	**β**	**S.E**.	**Wald**	**P**	**OR (95% CI)**
Age group (years)					
≤29	/	/	/	/	1
30–39	−0.33	0.17	3.66	0.06	0.72 (0.52–1.01)
40–49	−0.43	0.28	2.43	0.12	0.65 (0.38–1.12)
≥ 50	−1.32	0.45	8.72	0.003	0.27 (0.11–0.64)^*^
Sexual orientation disclosure	−0.46	0.17	7.66	0.006	1.58 (1.14–2.19)^*^
High–risk sexual behavior	0.39	0.17	5.20	0.02	1.47 (1.06–2.05)^*^
Entrapment	0.03	0.01	10.92	0.001	1.03 (1.01–1.05)^*^
Defeat	0.03	0.01	5.42	0.02	1.03 (1.004–1.05)^*^
Interpersonal needs	0.02	0.01	8.00	0.005	1.02 (1.01–1.03)^*^

* P < 0.05; OR, odds ratio.

The 882 MSM were randomly divided into two groups at a ratio of 3:1, which constituted the training set (*n* = 662) and the validation set (*n* = 220). The chi-square and non-parametric tests revealed no statistically significant differences between the training and validation sets for each variable, which indicated the baseline information was balanced between the two groups (Table 6).

**Table 6:** Descriptive analysis and test of difference between training set and validation set.

**Variables**	**Training set (*n* = 662)**	**Validation set (*n* = 220)**	**Z/χ^2^**	**P**
**Background factors**				
**Age group (years)**			2.84	0.42
≤29	288 (43.5)	83 (37.7)		
30–39	264 (39.9)	101 (45.9)		
40–49	76 (11.5)	24 (10.9)		
≥ 50	34 (5.1)	12 (5.5)		
**Education level**			0.08	0.96
Less than junior high school	100 (15.1)	34 (15.5)		
High school	126 (19.0)	40 (18.2)		
Uni. /tech./prof	436 (65.9)	146 (66.4)		
**Marital status**			0.14	0.93
Married	81 (12.2)	29 (13.2)		
Unmarried	550 (83.1)	181 (82.3)		
Divorced	31 (4.7)	10 (4.5)		
**Monthly income (RMB)**			1.10	0.78
≤3000	182 (27.5)	56 (25.5)		
3001–6000	230 (34.7)	73 (33.2)		
6001–12000	176 (26.6)	62 (28.2)		
≥12001	74 (11.2)	29 (13.2)		
**Local residence**			1.17	0.28
No	514 (77.6)	163 (74.1)		
Yes	148 (22.4)	57 (25.9)		
**Sexual orientation**			0.89	0.64
Heterosexuality	12 (1.8)	5 (2.3)		
Homosexuality	470 (71.0)	149 (67.7)		
Bisexuality	180 (27.2)	66 (30.0)		
**Sexual orientation disclosure**			0.27	0.60
Yes	387 (58.5)	133 (60.5)		
No	275 (41.5)	87 (39.5)		
**Triggering events**				
**Tobacco use**			4.18	0.12
No	451 (68.1)	152 (69.1)		
Sometimes	88 (13.3)	38 (17.3)		
Often/everyday	123 (18.6)	30 (13.6)		
**Alcohol use**			4.95	0.08
No	468 (70.7)	145 (65.9)		
Sometimes	178 (26.9)	73 (33.2)		
Often/everyday	16 (2.4)	2 (0.9)		
**Drug use**			0.30	0.59
No	317 (47.9)	110 (50.0)		
Yes	345 (52.1)	110 (50.0)		
**High–risk sexual behavior**			0.19	0.66
No	233 (35.2)	81 (36.8)		
Yes	429 (64.8)	139 (63.2)		
**Verbal harassment**			0.22	0.64
No	529 (79.9)	179 (81.4)		
Yes	133 (20.1)	41 (18.6)		
**Physical assault**			0.10	0.76
No	619 (93.5)	207 (94.1)		
Yes	43 (6.5)	13 (5.9)		
**Psychosocial factors**				
Entrapment	23 (18)	22 (17.75)	−0.85	0.40
Defeat	25 (19)	26 (17)	−0.11	0.91
Social support	42 (23)	41 (24)	−1.98	0.05
Interpersonal needs	61 (23)	64 (21)	−0.29	0.78

**Figure 2:**
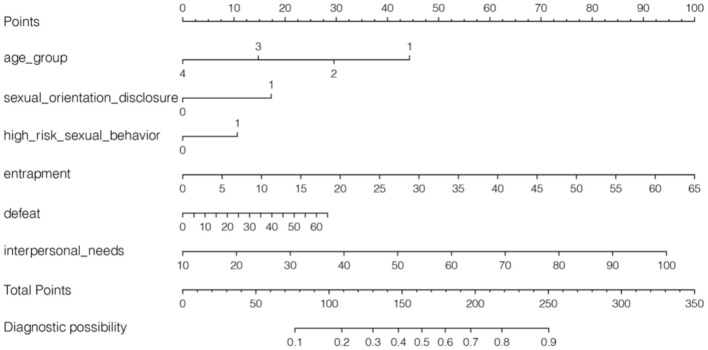
Developed nomogram of suicidal ideation based on the training set. Age group: 1 = ≤ 29; 2 = 30–39; 3 = 40–49; 4 = ≥ 50; sexual orientation disclosure, 1 = yes; 0 = no; high-risk sexual behavior, 1 = yes; 0 = no.

In the training set, a nomogram for predicting the risk of SI was developed using six predictors including age group, sexual orientation disclosure, high-risk sexual behavior, entrapment, defeat and interpersonal needs (Figure 2). The values of each predictor were assigned different scores, and a total score can be calculated to predict the probability of SI. Higher score indicated a higher probability of having SI.

**Figure 3:**
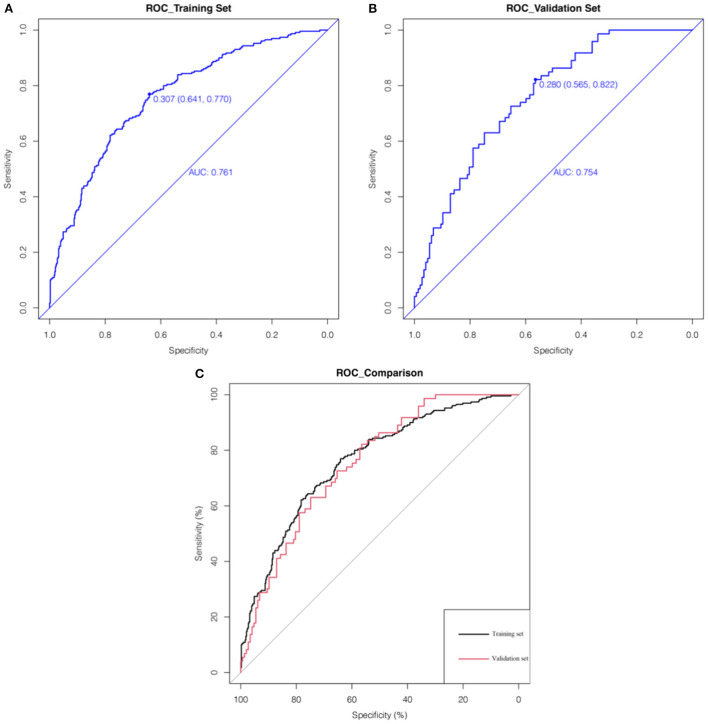
The pooled AUC of the ROC curves in the training set and validation set. The x axis represents the true negative rate of the risk prediction. The y axis represents the true positive rate of the risk prediction. The thick blue line represents the performance of the nomogram in the training set **(A)** and validation set **(B)**. The **(C)** merges the ROC curves of the two sets together, where the thick black and red lines represent the ROC curves of the training set and validation set, respectively.

**Figure 4:**
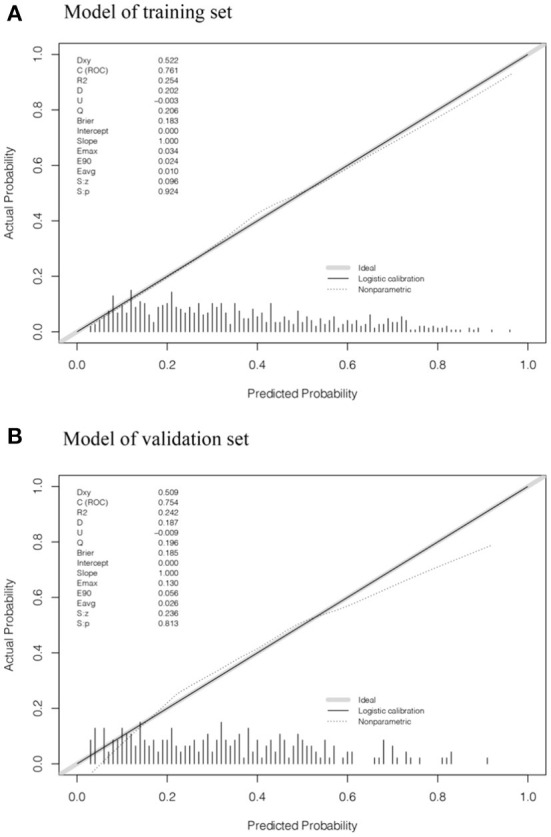
Calibration plots of suicidal ideation in the training set and validation set. **(A, B)** showed the calibration plots of the training set and validation set, respectively. The x axis and y axis represent the predicted probability and actual probability of MSM with suicidal ideation, respectively. The diagonal gray solid line represents a perfect prediction by an ideal model. The dotted line represents the nonparametric calibration of the nomogram. The black solid line represents the logistic calibration of the nomogram, of which a closer fit to the diagonal gray line represents a better prediction.

**Figure 5:**
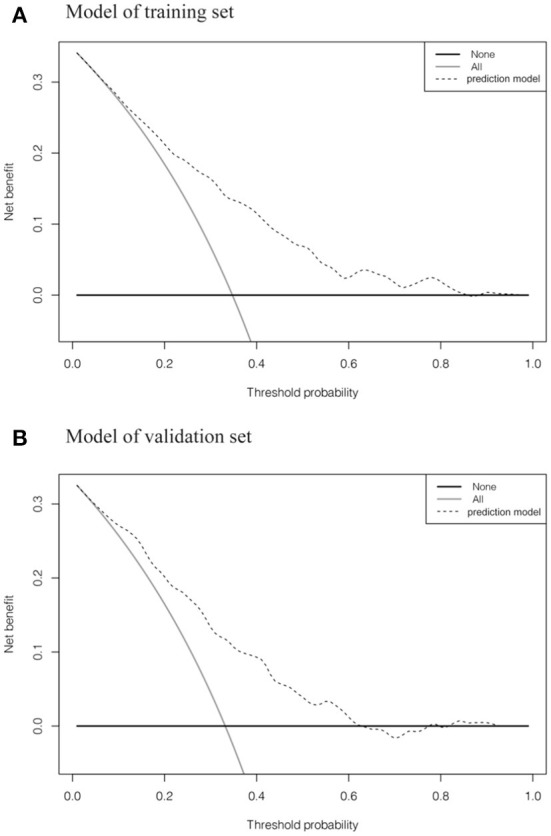
Decision curve analysis of suicidal ideation in the training set and validation set. **(A, B)** showed the decision curves of the training set and validation set, respectively. The y axis measures the net benefit. The dotted line represents the suicidal ideation incidence risk nomogram for MSM. The gray solid line represents the assumption that all patients are diagnosed as having suicidal ideation. The black solid line represents the assumption that no participants are identified as having suicidal ideation.

AUC values of ROC curves for nomogram were 0.761 (0.641–0.770) and 0.754 (0.565–0.822) in the training set and validation set, respectively, which indicated that the nomogram had favorable discrimination. And there was no statistical difference of the AUC values between the two sets (*P* > 0.05) (Figure 3). The calibration plots of the prediction models in both the training and validation sets fit well with the ideal model, and Hosmer-Lemeshow goodness-of-fit test showed that the predicted and actual probability were highly consistent (training set, *P* = 0.924; validation set, *P* = 0.813). The prediction models had good predictive performance (Figure 4). The DCA demonstrated that the threshold probability of prediction model in training set was 1–85% (Figure 5A), whereas in validation set was 1–63% (Figure 5B).

## 4. Discussion

This study showed that the lifetime prevalence of SI in the Chinese MSM is 34.4%. The value is generally consistent with the result reported in a systematic review (*P* = 34.79%) (4), but much higher than the Chinese general population and male population (9, 10). The lifetime prevalence of SI among MSM in China ranged from 10.6 to 26.0% (50). A study conducted in nine major Chinese cities surveyed 2,250 MSM, 26.01% of whom had considered suicide (34). Another study was conducted in four major cities in northeast China and the lifetime prevalence of SI was 18.3% (10). In other countries, the prevalence ranges from 20 to 50% (27). For example, 21% have had SI in the United States (51). The lifetime prevalence of SI in Nepal is 21.3% (52). In Bangkok, Thailand, 26.7% of MSM have had suicidal thoughts or have attempted suicide (53). In an Indian study, 45% of the participants reported SI (25). As can be seen, the reported prevalence varied greatly across countries or regions, possibly due to differences in measurement instruments or sample sources. However, the findings all support the same view that the prevalence of SI is significantly higher in MSM than in the general population.

The results showed that MSM aged 29 years and younger had a higher risk of SI than those aged 50 years and older. A study conducted in Shanghai came to similar conclusion that MSM under 25 years of age had a 5-fold higher risk of SI than MSM over 40 years of age (OR = 5.52, 95% CI: 1.26–24.18) (23). Concealing homosexual or bisexual orientation is a common coping strategy to avoid social stigmatization. Most Chinese MSM keep their homosexual or bisexual orientation a secret and do not seek help or support from their families or friends. Consistent with the findings of previous studies, disclosing or identifying as homosexual or bisexual may lead to more harm, shame, guilt, social exclusion and loss of support, thereby increasing the risk of suicide (54). Besides, MSM with high-risk sexual behaviors are more likely to develop SI. There is evidence that high-risk sexual behaviors are associated with psychological health problems (55). It has been suggested that MSM, when faced with multiple life stressors and coping with a variety of negative emotions, may adopt a range of avoidant or negative risk behaviors including high-risk sexual behavior, further leading to aggressive behaviors such as violence and suicide.

In this study, we identified three significant psychological factors that influence the development of SI among MSM. The higher the level of defeat and entrapment, and the more unmet interpersonal needs, the greater the likelihood of developing SI. The concept of entrapment and defeat derived from the social rank theory of depression. Defeat arises from the moment of losing social status or failed struggle. A sense of entrapment arises when individuals are not capable of escaping from a threat or pressure and remain trapped. Internal entrapment comes from the individuals' thoughts and feelings, while external entrapment comes from the outside environment or others (17). The concept of interpersonal needs derived from the Interpersonal Psychological Theory of Suicide (IPTS) developed by Thomas Joiner (56). The theory holds that negative emotion occurs when people perceive themselves as a burden or when they feel disconnected from society, and the risk of SI is strongest especially when the perception of burden and frustration of belonging coexist (57). In the IMV model, entrapment and defeat are core roles in the generation of SI, and interpersonal needs act as motivational moderators to facilitate the shift from entrapment to SI (16).

Previous research has used the IMV model to validate that defeat is significantly associated with entrapment, and that entrapment and interpersonal needs are significantly associated with SI in Chinese adolescent samples (58). A study found that defeat and entrapment can predict SI and entrapment was a mediator between defeat and SI, which supported the structure of the IMV model (59). Likewise, bisexual and other (e.g., pansexual)-identifying sexual minority people reported higher levels of IMV-related outcomes (e.g., internal entrapment, defeat) (60). Minority stress theory suggests that MSM, as sexual minorities, suffer from external prejudice, discrimination (distal stressors) and internal stressors such as internalized homophobia and secrecy (proximal stressors), resulting in a higher risk of mental health problems compared to the general population (50). MSM may be more prone to defeat and entrapment due to external social pressures (e.g., prejudice, discrimination, stigma, etc.) and internal psychological pressures (e.g., rejection of their own sexual orientation). When individuals are estranged from society and their basic interpersonal needs are not met, they may perceive that their presence places a burden on family, friends and/or society (61). The mental health problems could co-occur and interact resulting in the development of SI and suicide-related behaviors.

Prediction model can integrate various predictors to provide insight into the effects of predictors in the model. The development and validation of prediction model are important. Discrimination and calibration are significant indicators of a prediction model, and take in account the full range of predicted risks. Discrimination reflects the ability of the model to distinguish the subjects based on the nomogram prediction and observed outcomes. Calibration refers to the agreement between observed endpoints and predictions (45, 46). Besides, decision curve is used to assess the clinical utility, i.e., the ability to make better decisions with a model than without (62). And it is suggested to consider a range of thresholds when quantifying the clinical utility of a prediction model (48). In this study, we established a nomogram using six predictors (age group, sexual orientation disclosure, high-risk sexual behavior, entrapment, defeat and interpersonal needs) and validated its good predictive ability through discrimination, calibration and decision curves. We sought to develop and refine a nomogram that could predict SI in order to identify suicidal ideation in advance and implement interventions to reduce suicide-related behaviors among MSM.

## 5. Conclusion

The lifetime prevalence of SI among Chinese MSM is high. The nomogram can serve as a useful tool to predict the development of SI among MSM. We should pay more attention to MSM who disclose their sexual orientation and have high-risk sexual behaviors. Defeat, entrapment and interpersonal needs, as significant predictors of SI, can be measured to identify SI in advance. Early assessment of SI and the enhancement of psychosocial interventions are important to prevent suicide-related behaviors. Self-acceptance, reduction of stigma and burdensomeness, outside support, and reduction of prejudice and discrimination are important to protect the mental health of MSM. Future studies could incorporate more variables of interest to refine the prediction models to better guide behavioral and psychological intervention strategies in MSM population in practice.

## 6. Limitations

There were some limitations in this study. First, the study is a cross-sectional study and has limitations in the determination of causality. Therefore, further prospective studies are needed in the future to assess the causality between psychosocial variables and SI. Second, MSM are hard-to-reach and hidden populations, making it impossible to conduct random sampling, The snowball sampling method used in this study inevitably produced selection bias and sample representation problems. Expanding the sample size in subsequent studies could improve the sample representativeness to some extent. Third, the sample sizes of the four cities differed considerably and therefore cities were not included as independent variables in the statistical analysis. In the future, samples can continue to be collected across different cities to compare differences between cities. Finally, only one question was used in this study to measure the lifetime suicidal ideation of the participants, which is somewhat subjective. Multiple measurement instruments could be used in the future studies.

## Data availability statement

The raw data supporting the conclusions of this article will be made available by the authors, without undue reservation.

## Ethics statement

The study involving human participants was reviewed and approved by the Ethics Committee of the School of Public Health and Nursing, Shanghai Jiao Tong University School of Medicine. The participants provided their written informed consent to participate in this study.

## Author contributions

CX and ZW drafted the manuscript and performed statistical analyses. SL, HC, and DX were involved in the compilation of the questionnaire and data cleaning. YiC, HX, and YuC played major roles in the field survey. FH provided a lot of guidance on statistical analysis. YW, YoC, and JC made substantial contribution to the study design, data collection, the construction of the article framework, and the revision of the manuscript. All authors contributed the design of this research. All authors read and approved the final manuscript.

## References

[1] Bourne A Weatherburn P. Substance use among men who have sex with men: patterns, motivations, impacts and intervention development need. Sex Transm Infect. (2017) 93:342–46. 10.1136/sextrans-2016-052674 28400466

[2] Bagley C Tremblay P. Elevated rates of suicidal behavior in gay, lesbian, and bisexual youth. Crisis. (2000) 21:111–7. 10.1027//0227-5910.21.3.111 11265836

[3] Stall R Mills TC Williamson J Hart T Greenwood G Paul J. Association of co-occurring psychosocial health problems and increased vulnerability to HIV/AIDS among urban men who have sex with men. Am J Public Health. (2003) 93:939–42. 10.2105/AJPH.93.6.939 12773359 PMC1447874

[4] Luo Z Feng T Fu H Yang T. Lifetime prevalence of suicidal ideation among men who have sex with men: a meta-analysis. BMC Psychiatry. (2017) 17:406. 10.1186/s12888-017-1575-9 29268723 PMC5740861

[5] Klonsky ED May AM Saffer BY. Suicide, suicide attempts, and suicidal ideation. Annu Rev Clin Psychol. (2016) 12:307–30. 10.1146/annurev-clinpsy-021815-093204 26772209

[6] Nock MK Borges G Bromet EJ Alonso J Angermeyer M Beautrais A. Cross-national prevalence and risk factors for suicidal ideation, plans and attempts. Br J Psychiatry. (2008) 192:98–105. 10.1192/bjp.bp.107.040113 18245022 PMC2259024

[7] Hubers AAM Moaddine S Peersmann SHM Stijnen T Van Duijn E Van Der Mast RC. Suicidal ideation and subsequent completed suicide in both psychiatric and non-psychiatric populations: a meta-analysis. Epidemiol Psychiatr Sci. (2018) 27:186–98. 10.1017/S2045796016001049 27989254 PMC6998965

[8] Rm M. Suicidality and sexual orientation in five continents: Asia, Australia, Europe, North America, and South America. Int J Sex Gend Stud. (2002) 7:215–25. 10.1023/A:1015853302054

[9] Cao XL Zhong BL Xiang YT Ungvari GS Lai KY Chiu HF. Prevalence of suicidal ideation and suicide attempts in the general population of China: a meta-analysis. Int J Psychiatry Med. (2015) 49:296–308. 10.1177/0091217415589306 26060259 PMC4536918

[10] Mu H Li Y Liu L Na J Yu L Bi X. Prevalence and risk factors for lifetime suicide ideation, plan and attempt in chinese men who have sex with men. BMC Psychiatry. (2016) 16:117. 10.1186/s12888-016-0830-9 27129468 PMC4850688

[11] Wei D Wang X You X Luo X Hao C Gu J. Prevalence of depression, anxiety and suicide among men who have sex with men in china: a systematic review and meta-analysis. Epidemiol Psychiatr Sci. (2020) 29:E136. 10.1017/S2045796020000487 32536353 PMC7303796

[12] Klonsky ED May AM. Differentiating suicide attempters from suicide ideators: a critical frontier for suicidology research. Suicide Life Threat Behav. (2014) 44:1–5. 10.1111/sltb.12068 24313594

[13] O'connor R. Towards an Integrated Motivational–Volitional Model of Suicidal Behaviour, International Handbook of Suicide Prevention: Research, Policy and Practice. John Wiley & Sons, Ltd. (2011).

[14] Schotte DE Clum GA. Problem-solving skills in suicidal psychiatric patients. J Consult Clin Psychol. (1987) 55:49–54. 10.1037/0022-006X.55.1.49 3571658

[15] O'connor RC Nock MK. The psychology of suicidal behaviour. Lancet Psychiatry. (2014) 1:73–85. 10.1016/S2215-0366(14)70222-6 26360404

[16] O'connor RC Kirtley OJ. The integrated motivational-volitional model of suicidal behaviour. Philos Trans R Soc Lond B Biol Sci. (2018) 373:268. 10.1098/rstb.2017.0268 30012735 PMC6053985

[17] Gilbert P Allan S. The role of defeat and entrapment (arrested flight) in depression: an exploration of an evolutionary view. Psychol Med. (1998) 28:585–98. 10.1017/S0033291798006710 9626715

[18] O'connor R. C Portzky G. (2018). The relationship between entrapment and suicidal behavior through the lens of the integrated motivational-volitional model of suicidal behavior. Curr Opin Psychol 22, 12–17. 10.1016/j.copsyc.2017.07.021 30122271

[19] Remafedi G French S Story M Resnick MD Blum R. The relationship between suicide risk and sexual orientation: results of a population-based study. Am J Public Health. (1998) 88:57–60. 10.2105/AJPH.88.1.57 9584034 PMC1508407

[20] Koblin BA Chesney MA Husnik MJ Bozeman S Celum CL Buchbinder S. High-Risk behaviors among men who have sex with men in 6 us cities: baseline data from the explore study. Am J Public Health. (2003) 93:926–32. 10.2105/AJPH.93.6.926 12773357 PMC1447872

[21] Compton WM Jones CM. Substance use among men who have sex with men. N Engl J Med. (2021) 385:352–6. 10.1056/NEJMra2033007 34289278 PMC9169429

[22] Yu L Li Y Liu L Li S Na J An X. Association Of recent gay-related stressful events and emotional distress with suicidal behaviors over 12 months in Chinese men who have sex with men. Asia Pac Psychiatry. (2018) 10:12286. 10.1111/appy.12286 28636234

[23] Li R Cai Y Wang Y Sun Z Zhu C Tian Y. Psychosocial syndemic associated with increased suicidal ideation among men who have sex with men in Shanghai, China. Health Psychol. (2016) 35:148–56. 10.1037/hea0000265 26462059

[24] Wu YL Yang HY Wang J Yao H Zhao X Chen J. Prevalence of suicidal ideation and associated factors among hiv-positive msm in Anhui, China. Int J Std Aids. (2015) 26:496–503. 10.1177/0956462414544722 25060699

[25] Sivasubramanian M Mimiaga MJ Mayer KH Anand VR Johnson CV Prabhugate P. Suicidality, clinical depression, and anxiety disorders are highly prevalent in men who have sex with men in Mumbai, India: findings from a community-recruited sample. Psychol Health Med. (2011) 16:450–62. 10.1080/13548506.2011.554645 21749242 PMC3136931

[26] Rasmussen SA Fraser L Gotz M Machale S Mackie R Masterton G. Elaborating the cry of pain model of suicidality: testing a psychological model in a sample of first-time and repeat self-harm patients. Br J Clin Psychol. (2010) 49:15–30. 10.1348/014466509X415735 19302734

[27] Li R Cai Y Wang Y Gan F Shi R. Psychological pathway to suicidal ideation among men who have sex with men in shanghai, china: a structural equation model. J Psychiatr Res. (2016) 83:203–10. 10.1016/j.jpsychires.2016.09.002 27661416

[28] Balachandran VP Gonen M Smith JJ Dematteo RP. Nomograms in oncology: more than meets the eye. Lancet Oncol. (2015) 16:E173–80. 10.1016/S1470-2045(14)71116-7 25846097 PMC4465353

[29] Kan SK Chen NN Zhang YL. Predicting the risk of suicide attempt in a depressed population: development and assessment of an efficient predictive nomogram. Psychiatry Res. (2022) 310:114436. 10.1016/j.psychres.2022.114436 35190339

[30] Liang S Zhang J Zhao Q Wilson A Huang J Liu Y. incidence trends and risk prediction nomogram for suicidal attempts in patients with major depressive disorder. Front Psychiatry. (2021) 12:644038. 10.3389/fpsyt.2021.644038 34248696 PMC8261285

[31] Byeon H. Prediction of adolescent suicidal ideation after the covid-19 pandemic: a nationwide survey of a representative sample of Korea. Front Pediatr. (2022) 10:951439. 10.3389/fped.2022.951439 35958177 PMC9357914

[32] Luo Y Lai Q Huang H Luo J Miao J Liao R. Risk factor analysis and nomogram construction for predicting suicidal ideation in patients with cancer. BMC Psychiatry. (2022) 22:353. 10.1186/s12888-022-03987-z 35610595 PMC9128228

[33] Magnani R Sabin K Saidel T Heckathorn D. Review of sampling hard-to-reach and hidden populations for hiv surveillance. Aids. (2005) 19(Suppl. 2):S67–72. 10.1097/01.aids.0000172879.20628.e1 15930843

[34] Chen H Li Y Wang L Zhang B. Causes of suicidal behaviors in men who have sex with men in China: a national questionnaire survey. BMC Public Health. (2015) 15:91. 10.1093/oxfordhb/9780199366521.013.46 25885430 PMC4338620

[35] Xu C Yu X Tsamlag L Zhang S Chang R Wang H. Evaluating the validity and reliability of the Chinese entrapment scale and the relationship to depression among men who have sex with men in Shanghai, China. BMC Psychiatry. (2021) 21:328. 10.1186/s12888-021-03333-9 34215241 PMC8254295

[36] Hua T Suping W Ruijie G Zezhou W Yong C. Reliability and validity of defeat scale on anxiety and depression in medical students. J Shanghai Jiaotong Univer Sci. (2019) 39:84–88. (in chinese).

[37] Shangbin L Hao Y Chen X Jingwen D Xiaoyue Y Yong C. Validity and reliability of the chinese version of defeat scale in men who have sex with men. J Shanghai Jiaotong Univer. (2021) 4:1793–798. (in chinese).

[38] Van Orden KA Witte TK Gordon KH Bender TW Joiner TE. Jr. Suicidal desire and the capability for suicide: tests of the interpersonal-psychological theory of suicidal behavior among adults. J Consult Clin Psychol. (2008) 76:72–83. 10.1037/0022-006X.76.1.72 18229985

[39] Wang R Chen Y Hu F Wang Z Cao B Xu C. Psychometric properties of interpersonal needs questionnaire-15 for predicting suicidal ideation among migrant industrial workers In China. Int J Environ Res Public Health. (2021) 18:7583. 10.3390/ijerph18147583 34300033 PMC8306592

[40] Zimet GD Dahlem NW Zimet SG Farley GK. The multidimensional scale of perceived social support. J Person Asses. (1988) 52:30–41. 10.1207/s15327752jpa5201_2

[41] Yuan C Hongmei M Chen Z Yuling J Xiao W Jiaojiao C. Reliability and validity of chinese version of multidimensional scale of perceived social support in elderly people with chronic diseases. J Nurs. (2018) 25:5–8. (in chinese).15042639

[42] Stahlman S Grosso A Ketende S Pitche V Kouanda S Ceesay N. Suicidal ideation among msm in three west african countries: associations with stigma and social capital. Int J Soc Psychiatry. (2016) 62:522–31. 10.1177/0020764016663969 27515832

[43] Terwee CB Bot SD Boer D Van Der Windt MR Knol DA Dekker DL. Quality criteria were proposed for measurement properties of health status questionnaires. J Clin Epidemiol. (2007) 60:34–42. 10.1016/j.jclinepi.2006.03.012 17161752

[44] Pencina MJ D'agostino RB. Sr D'agostino RB. Jr Vasan RS. Evaluating the added predictive ability of a new marker: from area under the roc curve to reclassification and beyond. Stat Med. (2008) 27:157–72. 10.1002/sim.2929 17569110

[45] Van Calster B Mclernon DJ Van Smeden M Wynants L Steyerberg EW. Calibration: the achilles heel of predictive analytics. BMC Med. (2019) 17:230. 10.1186/s12916-019-1466-7 31842878 PMC6912996

[46] Steyerberg EW Vergouwe Y. Towards better clinical prediction models: seven steps for development and an ABCD for validation. Eur Heart J. (2014) 35:1925–31. 10.1093/eurheartj/ehu207 24898551 PMC4155437

[47] Shi R Zhang T Sun H Hu F. Establishment of clinical prediction model based on the study of risk factors of stroke in patients with type 2 diabetes mellitus. Front Endocrinol (Lausanne). (2020) 11:559. 10.3389/fendo.2020.00559 32982965 PMC7479835

[48] Vickers AJ Elkin EB. Decision curve analysis: a novel method for evaluating prediction models. Med Decis Making. (2006) 26:565–74. 10.1177/0272989X06295361 17099194 PMC2577036

[49] Vickers AJ Holland F. Decision curve analysis to evaluate the clinical benefit of prediction models. Spine J. (2021) 21:1643–48. 10.1016/j.spinee.2021.02.024 33676020 PMC8413398

[50] Sun S Pachankis JE Li X Operario D. Addressing minority stress and mental health among men who have sex with men (msm) In China. Curr HIV/AIDS Rep. (2020) 17:35–62. 10.1007/s11904-019-00479-w 31950336 PMC7050812

[51] Paul JP Catania J Pollack L Moskowitz J Canchola J Mills T. Suicide attempts among gay and bisexual men: lifetime prevalence and antecedents. Am J Public Health. (2002) 92:1338–45. 10.2105/AJPH.92.8.1338 12144994 PMC1447240

[52] Kohlbrenner V Deuba K Karki DK Marrone G. Perceived discrimination is an independent risk factor for suicidal ideation among sexual and gender minorities In Nepal. PLoS ONE. (2016) 11:E0159359. 10.1371/journal.pone.0159359 27437996 PMC4954730

[53] Guadamuz TE Mccarthy K Wimonsate W Thienkrua W Varangrat A Chaikummao S. Psychosocial health conditions and hiv prevalence and incidence in a cohort of men who have sex with men in Bangkok, Thailand: evidence of a syndemic effect. AIDS Behav. (2014) 18:2089–96. 10.1007/s10461-014-0826-8 24989128 PMC4198419

[54] Hidaka Y Operario D Tsuji H Takenaka M Kimura H Kamakura M. Prevalence of sexual victimization and correlates of forced sex in japanese men who have sex with men. PLoS ONE. (2014) 9:E95675. 10.1371/journal.pone.0095675 24802357 PMC4011701

[55] Mimiaga MJ Biello KB Robertson AM Oldenburg CE Rosenberger JG O'cleirigh C. High prevalence of multiple syndemic conditions associated with sexual risk behavior and HIV infection among a large sample of spanish- and portuguese-speaking men who have sex with men in Latin America. Arch Sex Behav. (2015) 44:1869–78. 10.1007/s10508-015-0488-2 26159862

[56] Joiner T. Why People Die By Suicide. Cambridge, MA: Harvard University Press (2006).

[57] Van Orden KA Witte TK Cukrowicz KC Braithwaite SR Selby EA. Joiner TEJr. The interpersonal theory of suicide. Psychol Rev. (2010) 117:575–600. 10.1037/a0018697 20438238 PMC3130348

[58] Li X Ren Y Zhang X Zhou J Su B Liu S. Testing the integrated motivational-volitional model of suicidal behavior in Chinese adolescents. Arch Suicide Res. (2021) 25:373–89. 10.1080/13811118.2019.1690607 32013796

[59] Owen R Dempsey R Jones S Gooding P. Defeat and entrapment in bipolar disorder: exploring the relationship with suicidal ideation from a psychological theoretical perspective. Suicide Life Threat Behav. (2018) 48:116–28. 10.1111/sltb.12343 28276599

[60] Rasmussen S Cramer RJ Mcfadden C Haile CR Sime VL Wilsey CN. Sexual orientation and the integrated motivational-volitional model of suicidal behavior: results from a cross-sectional study of young adults in the United Kingdom. Arch Suicide Res. (2021) 25:439–57. 10.1080/13811118.2019.1691693 31769357

[61] Meyer IH. Prejudice, social stress, and mental health in lesbian, gay, and bisexual populations: conceptual issues and research evidence. Psychol Bull. (2003) 129:674–97. 10.1037/0033-2909.129.5.674 12956539 PMC2072932

[62] Steyerberg EW Vickers AJ Cook NR Gerds T Gonen M Obuchowski N. Assessing the performance of prediction models: a framework for traditional and novel measures. Epidemiology. (2010) 21:128–38. 10.1097/EDE.0b013e3181c30fb2 20010215 PMC3575184

